# Perceived Risk of Genetically Modified Foods Among Residents in Xi’an, China: A Structural Equation Modeling Approach

**DOI:** 10.3390/ijerph16040574

**Published:** 2019-02-16

**Authors:** Wenjing Zhang, Jianhong Xue, Henk Folmer, Khadim Hussain

**Affiliations:** 1 Department of Agricultural Economics, Northwest A&F University, 3 Taicheng Road, Yangling 712100, China; zhangwj1105@126.com; 2 Department of Management, Xi’an University of Finance and Economics, 360 Changning Road, Xi’an 710100, China; 3 Faculty of Spatial Science, University of Groningen, Landleven 1, 9747 AD Groningen, The Netherlands; h.folmer@rug.nl; 4 Department of Economics, Mirpur University of Science & Technology (MUST), Mirpur 10250, AJK, Pakistan; hussain.eco@must.edu.pk

**Keywords:** perceived risks, structural equation modeling, genetically-modified food, China

## Abstract

This paper applies a structural equation modeling approach to study the formation of consumers’ perceived health risk of genetically modified (GM) foods based on a cross-sectional dataset of 508 consumers in Xi’an, China. The results indicate a high percentage of respondents who believe that GM foods might threaten human health. The estimated structural equation model shows that males, older people, respondents with higher income, those with better educational attainment, and those with family members who need special care have higher perceived risks of GM foods. Effective risk communication is necessary to provide consumers with scientific information about GM foods in order to facilitate their understanding of the actual risks of GM foods.

## 1. Introduction

Genetically modified (GM) food refers to the food produced from genetically modified organisms [1]. Since its first commercialization in 1996, the cultivation of GM foods worldwide has surged from 1.7 million hectares in 1996 to 185.1 million hectares in 2016 [2]. Scientists have warned a variety of potential risks related to human health and the environment potentially arise from GM foods. The risks concerning human health are the possibility to provoke allergic reactions, gene transfer, and outcrossing, whereas potential environmental risks include the possibility to introduce the engineered genes into wild populations, loss of biodiversity, and susceptibility of non-target organisms to the gene product [1,3,4].

Those risks concerning GM foods will be interpreted and proliferated by different interest parties and thus take strategic behavior to secure support for their preferred regulatory positions [5,6]. The regulations are used by the government to ensure the safety of GM foods for humans and the environment. If the products have been assessed to be safe for human health and the environment, the governments approve commercialization. In addition, regulations made by government agencies are proven to affect the whole food system and economic development to some extent. Wesseler (2017) revealed that the delayed approval of GM cowpea caused 33–46 million USD economic losses in one year [7]. Therefore, the regulation of GM technology and foods is a political process shaped not only by the costs and benefit of the interest groups but also by institutions and ideas [8,9,10]. It is necessary to study the ideas and attitudes of those interest groups related to GM technology and foods. Among those interest groups, the consumer plays an indispensable part. Research has demonstrated that the public’s attitude towards a technology or a food product is crucial to the implementation of the technological innovation and the commercialization of the food product [11,12,13,14]. It is also generally understood that people who consider a technology or food product risky are less likely to accept them [15,16,17,18]. However, the assessment of scientific risk is a rather complicated process, and ordinary people do not necessarily have the motivation and capacity to process the assessment. Next, individuals have different psychological, economic, and cultural characteristics, which might result in different reactions to even the same risk levels. Additionally, there is a chance that even a minor risk may arouse intense public concern and social impact, thus altering the perceived risks of the public [11,19,20,21]. In particular, previous studies have revealed that individuals are prone to cognitive biases which lead to differed objective and subjective risks [12,22,23,24,25]. The objective risks related to GM foods can be communicated by individuals to form their subjective risk perception. Due to the fact that the safety of GM foods is still uncertain and that individuals are confronted with uncertainties and contradictory information, their attitudes towards GM technology and foods are also based on subjective perception of risk [26,27,28]. Therefore, it is necessary to have a thoughtful understanding of consumers’ subjective perceived risks related to GM foods.

China is now ranked the eighth in the world for its planting area of GM crops, with a record of 2.8 million hectares of GM crops cultivated in 2016 [2]. China is also the largest importer of GM products. In 2016, the import of soybean in China was more than 850 million tons, accounting for 65% of the world’s total, among which more than 90% was GM [29,30]. Agricultural biotechnology is crucial in order to reduce the increasing reliance on imports, to enhance productivity and to improve China’s food self-sufficiency ratio. Following the Chinese government’s advancement of research and development of agricultural biotechnology, Chinese scientists have bred a variety of GM crops including cotton, rice, wheat, maize, soybean, potato, tomato, etc., some of which have been commercialized and others which are in-trial. In China’s 2006–2020 Long and Medium Term Scientific and Technological Development Plan, breeding new GM crops is one of its prioritized national programs. The central No.1 document in 2016 not only proposes further research and development of GM technology but also emphasizes the scientific communication and popularization of GM technology.

The surging cultivation and the mass import of GM foods have induced extensive debates between media and the scientific community over approved GM foods in China. GM foods are considered to be safe and contain several benefits as propagated by scientists who have been endeavoring to promote the planting and commercialization of GM foods. In contrast, the media emphasizes that the safety of GM food is not yet conclusive and that planting GM crops could lead to severe potential consequences [7]. There is no explicit conclusion about consumers’ attitudes towards GM foods as a result of the widespread dissemination of uncertainty and controversial information regarding GM foods [31,32,33,34]. For instance, in a survey conducted in Shanxi province, De Steur (2010) claimed that consumers were willing to pay an extra premium of 34% for GM rice with high folate content because rice with high folate content is good for pregnant women. On the contrary, Jin (2014) found that respondents in 13 rice consuming provinces in China required a 68% price discount to purchase GM rice. Meanwhile, Huang (2008) reported that more than 60% of residents in Hubei and Fujian province considered that GM and non-GM rice as perfect substitutes for each other. Based on a survey of 952 urban consumers in 15 Chinese provinces, Zheng et al. (2017) reported that the “golden rice” (Golden rice is a variety of nutritionally enhanced GM rice. In August 2012, the American Journal of Clinical Nutrition published a study testing the nutrition of golden rice by providing it to 68 Chinese children. The study was later found not having the parents’ consent and was retracted. There has been considerable debate about the safety of GM foods after the state media covered this event [35]) crisis has led to a decline in consumer’s willingness to buy GM rice. It was also well-documented that perceived risks were important determinants of the attitudes and purchase behaviors towards GM rice. In a survey of 250 respondents in Beijing, Kim and Chen (2013) reported that consumers’ perceived risk had a negative impact on their willingness to consume GM foods [36]. When consumers perceived GM foods are risky, they tend to develop a risk-reducing strategy, whereby they avoid the consumption of GM foods and are not willing to pay a high price for them.

Although these studies have explored the roles of perceived risk in consumers’ intentions to buy GM foods, to the best of our knowledge no studies have analyzed the mechanism under which Chinese consumers’ perceived risk of GM foods is formed. This study aims to fill this gap by investigating the determinants of perceived risk based on a survey of a sample of 508 respondents in Xi’an city, China. The remainder of the paper is organized as follows. The conceptual model is presented in Section 2, and the methodology and data are presented in Section 3. Section 4 discusses the empirical results, and Section 5 concludes the study.

## 2. The Conceptual Model

We present in this section the conceptual model to measure consumers’ perceived risk of GM foods. It describes the definitions of variables and the expected influences of the exogenous variables on the endogenous variable. We first discuss the endogenous variable, and then the exogenous variables.

### 2.1. Endogenous Variables: Perceived Risk of GM Foods

Perceived risk is defined as an individual’s assessment of hazards or dangers that might pose threats to his/her health and well-being [37]. There are two dimensions about the perceived risk of GM foods: (1) Perception of the possibility to harm health; and (2) perception of the severity of health risks. Following Kimenju and De Groote (2008), we consider respondents’ perception of risks relating to human health by asking them to indicate whether GM foods might damage human’s immune system, cause allergic reactions and gene mutation, increase antibiotic-resistant diseases, and cause infertility, on a 5-point scale from “very unlikely” to “very likely” [38]. Respondents’ attitude might be inconsistent depending on how the question is asked, that is, whether they are prompted or not during the survey [39].

### 2.2. Exogenous Variables

Control variables assumed to affect perceived risks of GM foods include socio-economic and demographic factors. The rationale for the inclusion of each factor is discussed below.

**Gender**

Males and females hold different attitudes toward a variety of health and food-related risks and concerns [40,41,42]. Women plan food and household purchases in most cases, thus they are more cautious about the food-related risks than men. In addition, previous studies have demonstrated that women develop more negative attitudes about biotechnology as a result of different life experiences and social responsibility compared with men [4,43]. Therefore, women might express a higher level of perceived risk than men. However, biotechnology, such as GM foods, is often associated with high productivity and lower price, which are two factors affecting women more than men [41]. Under this circumstance, women are prone to perceive less risk. Hence, the impact of gender on consumers’ perceived risk of GM foods can be positive or negative.

**Age**

Generally speaking, the elderly often stick to traditional lifestyles and are less likely to accept emerging technologies such as GM [44]. In contrast, younger individuals are more likely to tolerate new technology [24]. However, GM technology is a relatively new technology and is unfamiliar to many people, and older folks might regard it as a “high-level technology” and perceive fewer risks [45]. Meanwhile, compared to their older counterparts, younger people have better access to information about GM food and are more sensitive to the possible negative consequences on human health [44]. Consequently, there is a chance that younger people perceive more risks of GM foods than the elderly. Therefore, we cannot decide the impact of age on consumers’ perceived risk of GM foods.

**Education**

Education causes individuals to be more knowledgeable and competent to interpret a phenomenon [46]. Because GM is a new technology which is unfamiliar to many people, the ability to obtain and process information is necessary to form perceived risk. Hall and Moran (2006) found that education is an ideal way to give consumers knowledge and to make them informed about a more positive general attitude toward science and technology. They also found that respondents with postgraduate degrees perceived fewer risks related to GM foods compared to those with less education [47]. On the contrary, Grice and Lawrence (2003) pointed out that those with better educational attainment always seek more information related to GM foods, which stimulates negative attitudes towards GM foods [48]. Hence, the influence can be positive or negative on consumers’ perceived risk of GM foods.

**Income**

Previous studies revealed inconsistent results about the impact of income on consumers’ perceived risk of GM foods. Some studies showed that richer people perceive fewer risks than less affluent people because they are capable of purchasing foods that have no health and food safety problems [49]. However, some other studies demonstrated that higher income is associated with risk avoidance such that more affluent people perceived more risks than people with lower income [19]. On the contrary, Hwang (2005) reported that income did not have an impact on individuals’ perception of risk [50]. Hence, the relationship between respondents’ income and their perceived risks of GM foods is an empirical matter.

**Family Members Who Need Special Care (NFSC)**

The family members in need of special care refer to children, the elderly, pregnant women, and patients with chronic diseases. Respondents who live with people with these conditions have a higher risk perception from foods because they are more prone to food-borne illnesses [37,51]. Because the safety of GM foods has not been scientifically verified, respondents who have family members in need of special care might perceive more risks from GM food. Therefore, we assume that the number of respondents with family members in need of special care is positively associated with higher perceived risks. 

**Family Size**

Family size refers to the number of family members in a household. Compared to smaller families, larger families have more and better access to information about the risks of GM foods which could facilitate the development of a better understanding and judgment of GM foods [52]. Meanwhile, people in large families have higher perceived risk also because they have more family members to be concerned about. The number of family members can affect the budget in a household and the perceived cost of GM food. Therefore, the impact of family size on consumers’ perceived risks can be positive or negative.

**Poor Health Condition**

Consumers’ poor health condition is assumed to affect their perception of GM food risks. Consumers who have health conditions such as cardiovascular diseases, stomach diseases, food-related diseases, or other negative health conditions, will be more careful of the food they consume and may avoid foods that might pose a risk to their health. The safety of GM foods is still scientifically controversial. Hence, we assume that consumers’ poor health condition has a positive influence on their perceived risk of GM foods.

## 3. Methodology: Structural Equation Model (SEM)

The conceptual model will be estimated by a structural equation model (SEM). The conceptual model contains both latent variables (perceived risks) and observed variables (e.g., age and income). A latent variable refers to a phenomenon that exists but cannot be directly observed, such as intelligence, social status, and perception [53]. Nevertheless, a latent variable can be measured by a set of indicators, each of which has some explanatory power but none which measures a latent variable perfectly.

SEM provides a measurement model for latent variables and models their relationships within one system of equations [54]. SEM is composed of two parts, namely a measurement model and a structural model. The former represents the relationship between latent variables and their observables, whereas the latter specifies the relationships among latent variables and their exogenous determinants. The measurement models for the endogenous and exogenous variables are expressed in Equations (1) and (2), respectively.

(1) $$y=\Lambda_{y}\eta +\varepsilon$$

(2) $$x=\Lambda_{x}\xi +\delta$$

where y is a p×1 vector of observed variables to measure endogenous variables, x is a q×1 vector of observed variables to measure exogenous variables, η is a m×1 vector of latent endogenous variables, and *ξ* is a n×1 vector of latent exogenous variables. $\Lambda_{y}$ and $\Lambda_{x}$ are p×m and q×n matrices of factor loadings to be estimated. At last, ε and δ are p×1 and q×1 vectors of *i.i.d.* measurement errors, respectively.

The structural model is as follows:

(3) η=Bη+Γξ+ζ

where *B* is an m×m matrix of coefficients that represent the relationships among the latent endogenous variables, *Γ* is an m×n matrix of coefficients to model the association between the exogenous latent variables and the endogenous latent variables and *ζ* is an m×m vector of disturbances. However, this paper contains only one endogenous variable; the B matrix does not exist. The systems of equations described in Equations (1)–(3) can be rewritten as follows:

(4) $$\left[\begin{array}{c} \mathrm{PR}1\\ \vdots \\ \mathrm{PB}5 \end{array}\right]=\left[\begin{array}{c} \lambda_{1,1}\\ \lambda_{2,1}\\ \vdots \\ \lambda_{5,1} \end{array}\right]\times \left[\mathrm{PR}\right]+\left[\begin{array}{c} \varepsilon_{1}\\ ⁞\\ \varepsilon_{5} \end{array}\right]$$

(5) $$\left[\begin{array}{c} Gender\\ Age\\ Education\\ Income\\ FS\\ NFSC\\ HC \end{array}\right]=\left[\begin{array}{c} \begin{array}{c} 1\\ 0\\ 0 \end{array}\\ 0\\ 0\\ 0\\ 0 \end{array}\begin{array}{c} \begin{array}{c} 0\\ 1\\ 0 \end{array}\\ 0\\ 0\\ 0\\ 0 \end{array}\begin{array}{c} \begin{array}{c} 0\\ 0\\ 1 \end{array}\\ 0\\ 0\\ 0\\ 0 \end{array}\begin{array}{c} \begin{array}{c} 0\\ 0\\ 0 \end{array}\\ 1\\ 0\\ 0\\ 0 \end{array}\begin{array}{c} \begin{array}{c} 0\\ 0\\ 0 \end{array}\\ 0\\ 1\\ 0\\ 0 \end{array}\begin{array}{c} \begin{array}{c} 0\\ 0\\ 0 \end{array}\\ 0\\ 0\\ 1\\ 0 \end{array}\begin{array}{c} \begin{array}{c} 0\\ 0\\ 0 \end{array}\\ 0\\ 0\\ 0\\ 1 \end{array}\right]\times \left[\begin{array}{c} Gender\\ Age\\ Education\\ Income\\ FS\\ NFSC\\ HC \end{array}\right]$$

(6) $$\left[PR\right]=\left[\begin{array}{cc} \gamma_{11} & \gamma_{12} \end{array}\begin{array}{ccc} \gamma_{13} & \gamma_{14} & \gamma_{15} \end{array}\right]\times \left[\begin{array}{c} Gender\\ Age\\ Education\\ Income\\ FS\\ NFSC\\ HC \end{array}\right]+\left[\zeta_{1}\right]$$

## 4. Empirical Results

### 4.1. The Survey

Our data was based on a survey conducted in Xi’an city, Shaanxi province in Northwestern China. The total population of Xi’an city was 8.63 million in 2014, scattered in 10 municipal districts and 3 counties [55]. Respondents were randomly approached at supermarkets, wholesale markets, and parks located in those districts and counties. The questionnaire was developed to elicit respondents’ sociodemographic characteristics, awareness of GM foods, and perceived risks towards GM foods. Face-to-face interviews were conducted by interviewers. Prior to the interviews, a preliminary survey was conducted to test the questionnaire in order to remove unclear formulations. The interviewers were a group of Master’s and Ph.D. students from the College of Economics and Management, Northwest A&F University, Shaanxi province. The survey was carried out between May and June 2015.

### 4.2. Descriptive Statistics 

Table 1 describes the interviewee’s socio-demographic characteristics. It can be seen that there are more female (55.1%) than male respondents (44.9%) (Male = 0, Female = 1). This is different from the census data (Female: 48.4% VS Male: 51.6%), since women are often the nurturers and career providers of the family who regularly shop at farmers’ markets or supermarkets. The mean age is approximately 34, indicating that the interviewees are relatively young (shoppers are often younger adults). Their educational attainment is high: 36.2% of the respondents had bachelor’s degrees and above. In terms of income, the statistics show that 37.8% of the respondents had a monthly income of 1000–3000 Yuan, and another 32.7% had a 3000–5000 Yuan monthly income, consistent with the average monthly per capita income of 4,343 Yuan reported in the census data [55]. Next, 26.3% of respondents had at least one family member who was elderly (more than 60 years old); 9.97% of citizens are more than 65 years old according to the census data, and 28.1% had at least one child younger than 12 years old (14.1% of the citizens are less than 14 years old based on the statistical data), 3.0% lived with pregnant women, 4.4% had infants younger than 6 months, and 7.7% had family members with chronic diseases.

The explicit statements and their summary statistics for perceived risks are presented in Table 2. Perceived risks of GM foods are relatively high with 33.66% and 12.99% of the respondents believing that it is “likely” and “totally likely” that GM foods might damage the human immune system, respectively. Around 40% of the respondents considered it “likely” or “totally likely” that GM foods might cause allergic reactions and gene mutations for humans, and more than 40% agreed that GM foods could cause antibiotic-resistant and infertility. On the contrary, only 10% believed it impossible that GM foods could cause risks for human health, suggesting that respondents had limited understanding of the possible negative effects of GM foods on human health.

### 4.3. The Estimated SEM

The measurement and structural models, as specified in Equations (4)–(6), are presented in Tables 4 and 5, respectively. Because some observed variables are ordinal or discrete, our model is estimated using the maximum likelihood method based on a matrix of polychoric correlations [54]. Two models have been estimated: The initial model, which includes all exogenous variables, and a final model, which was obtained by following a stepwise procedure where the most insignificant variable drops out from the model one by one until all variables have significant coefficients.

We first discuss the goodness-of-fit measures in Table 3. The fit statistics for the initial model meet the thresholds, indicating good fits suggested by Hooper et al. (2008): $\chi^{2}$/DF (2.42), GFI (0.97), AGFI (0.94), CFI (0.98), IFI (0.98), RMSEA (0.053), and SRMR (0.024). Similarly, the final model also has a reasonably well model fit: $\chi^{2}$/DF (2.81), GFI (0.97), AGFI (0.94), CFI (0.98), IFI (0.98), RMSEA (0.0537), and SRMR (0.026) [56].

Next, we turn to the measurement model presented in Table 4. Since the measurement models for the initial and final model are very similar, we only discuss the results for the initial model. It can be seen that perceived risks were well measured. The standardized factor loadings for observables ranged between 0.73 and 0.83 and all were above 0.4, demonstrating that all indicators had satisfactory validity. The reliability measures of the indicators were reasonable as well. Looking closer, the R2 for the perceived health risks, PR1-PR5, were all above 0.50, indicating relatively high reliability.

Table 5 shows the estimated coefficients and standard errors (in parenthesis) for the structural model. We first discuss the initial model, then the final model. Gender has a positive impact (0.15, *p* < 0.01) on perceived risks, which means males had higher risk perception than their female counterparts. The possible explanation is that there is an increasing number of males taking care of their families, and they pay more attention to dietary health and are more sensitive to biotechnology than females. Hence, males perceived more risks of GM food than women. Age positively and significantly affects perceived risks (0.22, *p* < 0.01), indicating that the elderly considered GM foods as riskier than younger adults. This suggests that older adults tend to have lower acceptability of new things. Education has a positive impact on perceived risks (0.10, *p* < 0.10), meaning that education does not play much of a role. This is related to respondents’ employment field, and more highly educated people who worked in areas such as biotechnology perceived fewer risks of GM foods. Next, income has a positive influence on perceived risks (0.10, *p* < 0.05), suggesting that more affluent respondents perceived higher risks. Compared to less affluent people, those with higher income have fewer budget constraints, and thus they are capable of buying less risky foods. Family size did not have a significant influence on perceived risks, suggesting that larger family size does not promote a higher perceived risk. The potential reason is that GM food is not a frequent topic for discussion among family members. In addition, larger families take more consideration of the food price, and the price of GM foods is often lower than conventional foods, so they perceive fewer risks. NFSC exerts a positive and significant influence on perceived risks (0.13, *p* < 0.05), indicating that individuals with someone in need of special care are cautious about GM foods and thus they perceived more risks.

We now turn to the final model. The standardized coefficients for the final model are largely in line with the numbers in the initial model. The only difference is that the insignificant coefficient for education (0.10, *p* > 0.10) became significant in the final model (0.11, *p* < 0.10). This means that respondents with higher education perceived more risks of GM foods. The rationale is that individuals with better educational attainment are prone to believe GM foods might threaten their health. Additionally, the health condition of individuals does not carry much weight when it comes to influencing perceived risks (0.02, *p* > 0.10). A possible explanation is that compared to healthy respondents, unhealthy respondents were not necessarily more likely to consider GM foods as potential causes to their illnesses.

## 5. Summary and Conclusions

China is seeing a surge in the cultivation of GM crops, imports of GM foods, as well as research and development of GM technology. Previous studies have explored the roles of perceived risks in consumers’ intentions to buy GM foods but have neglected the mechanisms under which consumers’ perceived risks are formed. This paper aims to analyze consumers’ perceived risks of GM foods and their determinants, based on a sample of 508 consumers in Xi’an city, Shaanxi province, China. The main results are as follows.

First, the percentage of respondents who believed that GM foods might threaten human health and the environment was much higher than those respondents who believed not so. Meanwhile, a large portion (more than 40%) of respondents had limited understanding of the risks of GM foods. The approval process of GM technology and foods affects the whole food system and has an impact on economic development. The interest groups provide different explanations of the potential risks with regard to GM foods, so consumers might be confronted with contradictory information. Consumers’ opinions play a pivotal role in policy development, and therefore, effective risk communication is necessary to provide scientific and objective information about GM foods to consumers in order to facilitate their understanding of the physical risks of GM foods. China has the largest number of mobile phone users in the world, with 1.8 billion mobile phones sold in the fourth quarter of 2018 [57], and 89% of Chinese internet users use smartphones to go online [58]. One effective way to facilitate a better understanding of GM foods would be to develop smartphone apps combined with social media platforms (e.g., WeChat) to communicate this information to the masses. Furthermore, foods processed with GM materials and ingredients need to be clearly labeled. Under this circumstance, consumers can make their own judgments about GM foods and can make their own choices.

Second, the results of the structural equation model showed that gender, age, education, income, and NFSC had significant influences on the perceived risks of GM foods. These results suggested that the communication of risks relating to GM foods would be more effective if these specific groups are targeted. In particular, the consumers who had family members in need of special care were more concerned about the risks. This fact agrees with the results that respondents’ perceived risks were more characterized by perceived health risks. 

## Figures and Tables

**Table 1:** Respondents’ characteristics.

Variables	Min.	Max.	Mean	S.D.
Gender	0	1	0.45	0.5
Age	15	86	34.41	10.95
Education	%	Income (Yuan/Month) ≤1000 1001–3000 3001–5000 5001–7000 ≥7001		% 21.46 37.8 32.68 5.12 2.95
Primary school or none	6.69			
Middle school	12.2			
High school/Vocational School	20.87			
Junior college	23.03			
Bachelor’ degree	31.69			
Master’ degree and above	5.57			
NFSC	%			
Pregnant woman	2.96			
Infant younger than 6-month old	4.44			
Patient with chronic diseases	7.73			
Elderly	26.32			
Child younger than 12	28.13			
None	30.43			

**Table 2:** Frequency distribution of responses to perceived risks statements.

Statements	Notations	Totally Unlikely	Unlikely	Unsure	Likely	Totally Likely
GM foods might damage our immune system.	PR1	1.38%	7.09%	44.88%	33.66%	12.99%
GM foods might cause allergic reactions.	PR2	1.38%	5.12%	52.17%	29.92%	11.42%
GM foods might cause gene mutation.	PR3	1.97%	9.06%	48.23%	27.95%	12.80%
GM foods might increase antibiotic-resistant diseases	PR4	1.38%	6.89%	50.59%	28.74%	12.40%
GM foods might cause infertility.	PR5	1.57%	6.89%	50.00%	29.33%	12.20%

**Table 3:** The goodness-of-fit measures.

Model-Fit Statistics	Initial Model	Final Model	Critical Values
$\chi^{2}$/DF	2.42	2.81	<3
Goodness-of-fit index (GFI)	0.97	0.97	>0.90
Adjusted goodness-of-fit index (AGFI)	0.94	0.94	>0.90
Comparative fit index (CFI)	0.98	0.98	>0.90
Incremental fit index (IFI)	0.98	0.98	>0.90
Root mean square error of approximation (RMSEA)	0.053	0.057	<0.05
Standardized root mean square residual (SRMR)	0.024	0.026	<0.08

**Table 4:** Estimated measurement model.

	Initial Model			Final Model		
Indicators	Standardized Coefficients	S.E.	R^2^	Standardized Coefficients	S.E.	R^2^
PR1	0.83	-	0.9	0.83	-	0.69
PR2	0.83 ***	0.05	0.70	0.83 ***	0.05	0.70
PR3	0.77 ***	0.05	0.59	0.77 ***	0.05	0.59
PR4	0.73 ***	0.05	0.53	0.73 ***	0.05	0.53
PR5	0.76 ***	0.05	0.58	0.76 ***	0.05	0.58

**Table 5:** The estimated structural model.

Perceived Risks (PR)	Initial Model	Final Model
Gender	0.15 *** (0.05)	0.15 *** (0.05)
Age	0.22 *** (0.06)	0.21 *** (0.06)
Education	0.10 (0.06)	0.11 * (0.06)
Income	0.10** (0.05)	0.11 ** (0.05)
Family size	−0.07 (0.05)	
NFSC (Needs for special care)	0.13 ** (0.05)	0.10 ** (0.05)
Health condition	0.02 (0.05)	

## References

[1] WHO (2014). Frequently Asked Questions on Genetically Modified Foods.

[2] ISAAA (2017). 2017 Global Status of Commercialized Biotech/GM Crops in 2017.

[3] Verma C., Nanda S., Singh R., Singh R., Mishra S. (2011). A Review on Impacts of Genetically Modified Food on Human Health. Open Nutraceut. J..

[4] Gaskell G., Alum N., Wagner W., Kronberger N., Torgensern H., Hampel J., Bardes J. (2004). GM foods and the misperception of risk perception. Risk Anal..

[5] Herring R., Paarlberg R. (2016). The political economy of biotechnology. Ann. Rev. Res. Econ..

[6] Shao Q., Punt M., Wesseler J. (2018). New Plant Breeding Techniques Under Food Security Pressure and Lobbying. Front. Plant Sci..

[7] Wesseler J., Smart R.D., Thomson J., Zilberman D. (2017). Foregone benefits of important food crop improvements in Sub-Saharan Africa. PLoS ONE.

[8] Graff G.D., Hochman G., Zilberman D. (2009). The political economy of agricultural biotechnology policies. AgBioForum.

[9] Wesseler J., Zilberman D. (2014). The Economic Power of the Golden Rice Opposition. Environ. Dev. Econ..

[10] Smart R.D., Blum M., Wesseler J. (2015). EU Member States’ Voting for Authorizing Genetically Engineered Crops: A Regulatory Gridlock. Ger. J. Agric. Econ..

[11] Verbeke W., Frewer L.J., Scholderer J., De Brabander H.F. (2007). Why consumers behave as they do with respect to food safety and risk information. Anal. Chim. Acta.

[12] Bearth A., Cousin M., Siegrist M. (2014). The consumer’s perception of artificial food additives: Influences on acceptance, risk and benefit perceptions. Food Qual. Preference.

[13] Lobb A.E., Mazzocchi M., Traill W.B. (2007). Modelling risk perception and trust in food safety information within the theory of planned behavior. Food Qual. Preference.

[14] Satterfield T., Kandlikar M., Beaudrie C.E., Conti J., Harthorn B.H. (2009). Anticipating the perceived risk of nanotechnologies. Nat. Nanotechnol..

[15] Wilson C., Evan G., Leppard P., Syrette J. (2004). Reaction to genetically modified food crop and how perception of risks and benefits influence consumers’ information gathering. Risk Anal..

[16] Costa-Font M., Gil J.M. (2009). Structural equation modeling of consumer acceptance of genetically modified (GM) food in the Mediterranean Europe: A cross country study. Food Qual. Preference.

[17] Van Kleef E., Houghton J.R., Krystallis A., Pfenning U., Rowe G., Van Dijk H., Van der Lans I.A., Frewer L.J. (2007). Consumer evaluations of food risk management quality in Europe. Risk Anal..

[18] Frewer L.J., Bergmann K., Brennan M., Lion R., Meertens R., Rowe G., Siegrist M., Vereijken C.M. (2011). Consumer response to novel agri-food technologies: Implications for predicting consumer acceptance of emerging food technologies. Trends Food Sci. Technol..

[19] Frewer L.J. (2017). Consumer acceptance and rejection of emerging agrifood technologies and their applications. Eur. Rev. Agric. Econ..

[20] Gupta N., Fischer A.R.H., Frewer L.J. (2011). Socio-psychological determinants of public acceptance of technologies: A review. Public Underst. Sci..

[21] Kasperson R.E., Renn O., Slovic P., Brown H.S., Emel J., Goble R., Kasperson J.X., Ratick S. (1988). The social amplification of risk: A conceptual framework. Risk Anal..

[22] Slovic P. (1999). Trust, emotion, sex, polities, and science: Surveying the risk-assessment battlefield. Risk Anal..

[23] Frewer L.J., Howard C., Aaron I. (1998). Consumers acceptance of transgenic crops. Pestic. Sci..

[24] Antonopoulou L., Papadas C.T., Targoutzidis A. (2009). The Impact Of Socio-Demographic Factors And Political Perceptions On Consumer Attitudes Towards Genetically Modified Foods: An Econometric Investigation. Agric. Econ. Rev..

[25] van der Linden S. (2015). The social-psychological determinants of climate change risk perceptions: Towards a comprehensive model. J. Environ. Psychol..

[26] Prati G., Pietrantoni L., Zani B. (2012). The prediction of intention to consume genetically modified food: Test of an integrated psychosocial model. Food Qual. Preference.

[27] Amin L., Azad M., Abul K., Gausmian M.H., Zulkifli F. (2014). Determinants of Public Attitudes to Genetically Modified Salmon. PLoS ONE.

[28] Bawa A.S., Anilakumar K.R. (2013). Genetically modified foods: Safety, risks and public concerns—A review. J. Food Sci. Technol..

[29] Zheng Z., Gao Y., Zhang Y., Henneberry S. (2017). Changing attitudes toward genetically modified foods in urban China. China Agric. Econ. Rev..

[30] ISAAA (2016). 2016 Global Status of Commercialized Biotech/GM Crops: 2016.

[31] Jin J., Wailes E.J., Dixon B.L., Nayga R.M., Zheng Z. Consumer acceptance and willingness to pay for genetically modified rice in China. Proceedings of the 2014 AAEA Annual Meeting.

[32] De Steur H., Gellynck X., Storozhenko S., Liqun G., Lambert W., Van Der Straeten D., Viaene J. (2010). Willing to accept and purchase genetically modified rice with high folate content in Shanxi province, China. Appetite.

[33] Zhang X., Huang J., Qiu H., Huang Z. (2010). A consumer segmentation study with regards to genetically modified food in urban China. Food Policy.

[34] Huang J., Hu R., Rozelle S., Pray C. (2008). Genetically Modified Rice, Yields, and Pesticides: Assessing Farm-Level Productivity Effects in China. Econ. Dev. Cult. Chang..

[35] Dubock A. (2014). The politics of Golden Rice. GM Crops Food.

[36] Kim R.B., Chen H. (2013). Chinese consumers’ choice for genetically modified (GM) food: Implication for food risk policy in China. Act. Probl. Econ..

[37] Adeola F.O. (2007). Nativity and Environmental Risk Perception: An Empirical Study of Native-Born and Foreign-Born Residents of the USA. Hum. Ecol. Rev..

[38] Kimenju S.C., De Groote H. (2008). Consumer willingness to pay for genetically modified food in Kenya. Agric. Econ..

[39] Marks L.A., Kalaitzandonakes N., Vickner S.S. (2003). Evaluating Consumer Response to GM Foods: Some Methodological Considerations. Curr. Agric. Food Resour. Issues.

[40] Hamilton L.C. (1985). Concern about toxic wastes: Three demographic predictors. Sociol. Perspect..

[41] Moerbeek H., Casimir G. (2005). Gender Differences in Consumers’ Acceptance of Genetically Modified Foods. Int. J. Consum. Stud..

[42] Simon R.M. (2010). Gender differences in knowledge and attitude towards biotechnology. Public Underst. Sci..

[43] Qin W., Brown J.L. (2007). Public Reactions to Information about Genetically Engineered Foods: Effects of Information Formats and Male/Female Differences. Public Underst. Sci..

[44] Veeman M., Adamowicz W., Hu W. (2005). Risk Perceptions, Social Interactions and the Influence of Information on Social Attitudes to Agricultural Biotechnology.

[45] Chen M., Li H. (2007). The consumer’s attitude toward genetically modified foods in Taiwan. Food Qual. Preference.

[46] Tang J.J., Folmer H., Xue J.H. (2013). Estimation of awareness and perception of water scarcity among farmers in the Guanzhong Plain, China, by means of a structural equation model. J. Environ. Manag..

[47] Hall C., Moran D., Investigating G.M. (2006). Perceptions: A survey of anti-GM and environmental campaign group members. J. Rural Stud..

[48] Grice J., Lawrence G. Consumer surveys of biotechnology: Asking the questions until we get the answers we want OR Empowering the public to express their opinion. Presented at the Agrifood Research Network Conference.

[49] Dosman D.M., Adamowicz W.L., Hrudey S.E. (2001). Socioeconomic Determinants of Health- and Food Safety-Related Risk Perceptions. Risk Anal..

[50] Hwang Y.J., Roe B., Teisl M.F. (2005). An empirical analysis of united states consumers’ concerns about eight food production and processing technologies. AgBioForum.

[51] Boccaletti S., Nardellab M. (2001). Consumer willingness to pay for pesticide-free fresh fruit and vegetables in Italy. Int. Food Agribus. Manag. Rev..

[52] Xu P., Zheng S., Motamed M. (2010). Perceived risks and safety concerns about fluid milk among Chinese college students. Agric. Econ..

[53] Oud J.H., Folmer H. (2008). A structural equation approach to models with spatial dependence. Geogr. Anal..

[54] Jöreskog K.G., Sörbom D. (2001). LISREL 8: User’s Reference Guide.

[55] National Bureau of Statistics of the People’s Republic of China (2015). Shaanxi Statistical Yearbook.

[56] Hooper D., Coughlan J., Mullen M. (2008). Structural Equation Modelling: Guidelines for Determining Model Fit. Electron. J. Bus. Res. Methods.

[57] National Bureau of Statistics of the People’s Republic of China (2018). China Statistical Yearbook 2018.

[58] Jing M. 89% of Chinese Internet Users Use Smartphone to Go Online. China Daily. http://www.chinadaily.com.cn/business/tech/2015-07/23/content_21388108.htm.

